# KIRSCHNER WIRES *VERSUS* SCREW FIXATION FOR LATERAL HUMERAL CONDYLE FRACTURES IN CHILDREN: A SYSTEMATIC REVIEW AND META-ANALYSIS

**DOI:** 10.1590/1413-785220263403e300984

**Published:** 2026-06-22

**Authors:** Jessiel Gonçalves de Souza, Adriano Assis Nascimento, Marina Garcia Seike, Fabio Akio Takihi, Israel Quirino Cavalcante, Eiffel Tsuyoshi Dobashi

**Affiliations:** 1 Universidade Federal de Sao Paulo, Escola Paulista de Medicina, Departamento de Ortopedia e Traumatologia, Grupo Ortopedia Pediatrica, Sao Paulo, SP, Brazil.

**Keywords:** Humeral Fractures, Fracture Fixation, Internal, Elbow Joint, Bone Screws, Kirschner Wires, Pediatrics, Fraturas do Úmero, Fixação Interna de Fraturas, Articulação do Cotovelo, Parafusos Ósseos, Fios de Kirschner, Pediatria

## Abstract

**Objective:**

Lateral condyle fractures of the humerus are the second most common elbow fracture in children. Both Kirschner wires (K-wires) and cannulated screws are widely used for fixation, but the optimal technique remains debated. This study aimed to compare these methods in terms of functional outcomes and complications.

**Material and Methods:**

A systematic review and meta-analysis were conducted in accordance with PRISMA guidelines.

**Results:**

Thirteen comparative studies involving 1,230 pediatric patients were included. Outcomes were analyzed using random-effects models, with risk ratios (RRs) and 95% confidence intervals (CIs). Meta-regression and prediction intervals (PIs) were applied to explore heterogeneity. Screw fixation significantly reduced the risk of postoperative infection (RR = 0.27; 95% CI: 0.12–0.60) and was associated with better functional outcomes (RR = 1.09; 95% CI: 1.01–1.18). No significant differences were observed in overall complications (RR = 0.89; 95% CI: 0.66–1.20), lateral overgrowth, range of motion limitation, or nonunion. Meta-regression did not identify age or fracture severity as effect modifiers.

**Conclusion:**

Cannulated screw fixation may be preferable for reducing infection and improving outcomes, but individualized clinical decisions and further high-quality research remain essential. ***Level of Evidence III; Systematic Review.***

## RESUMO

**Objetivo:**

A fratura do côndilo lateral do úmero é a segunda mais comum do cotovelo da criança. Os fios de Kirschner e os parafusos canulados são amplamente utilizados para sua fixação, mas o dispositivo ideal ainda é discutível. Este estudo teve como objetivo comparar esses métodos considerando os resultados funcionais e as complicações.

**Material e Métodos:**

Foi realizada uma revisão sistemática e uma metanálise, seguindo as diretrizes PRISMA.

**Resultados:**

Treze estudos comparativos envolvendo 1.230 pacientes pediátricos foram incluídos. Os resultados foram analisados usando modelos de efeitos aleatórios, com razões de risco (RRs) e intervalos de confiança (ICs) de 95%. Meta-regressão e intervalos de predição (PIs) foram aplicados para explorar a heterogeneidade. A fixação com parafuso reduziu significativamente o risco de infecção pós-operatória (RR = 0,27; IC 95%: 0,12–0,60) e foi associada a melhores resultados funcionais (RR = 1,09; IC 95%: 1,01–1,18). Nenhuma diferença significativa foi observada considerando: complicações gerais (RR = 0,89; IC 95%: 0,66–1,20), supercrescimento lateral, limitação da amplitude de movimento ou não-união. A meta-regressão não identificou idade ou gravidade da fratura como modificadores de efeito.

**Conclusão:**

A fixação com parafuso canulado é preferencial para reduzir infecções e melhorar os resultados, mas decisões clínicas individualizadas e pesquisas futuras de alta qualidade continuam sendo essenciais. ***Nível de Evidência III; Revisão Sistemática.***

## INTRODUCTION

Fractures of the lateral condyle of the humerus represent the second most common type of elbow fracture in the pediatric population, accounting for approximately 12% to 20% of these injuries. These fractures predominantly affect children aged 5 to 10 years and are typically the result of a fall with the hand extended, leading to an avulsion injury at the lateral condyle due to traction on the extensor muscles.^1^

Involvement of the growth plate increases the risk of disturbances to this structure, while intra-articular extension may lead to bone incongruence if not managed properly. Consequently, accurate diagnosis and appropriate treatment are critical to prevent complications such as non-union, malunion, angular deformities, and long-term functional impairment.^2^

Surgical intervention is generally indicated for fractures that exhibit displacement greater than 2 mm, especially when there is disruption of the articular surface or instability on stress testing.^2^ The two main methods of surgical fixation are the use of Kirschner wires (K-wires) and cannulated screws. K-wire fixation is a traditional technique that involves the percutaneous insertion of wires to stabilize the fracture, often requiring prolonged immobilization and carrying a risk of pin-tract infections.^3^ On the other hand, cannulated screw fixation provides stable internal stabilization, allowing for early mobilization and reducing the risk of superficial infections.^4^ However, screw fixation generally requires a second surgical procedure for removal, similar to K-wires. The choice between these methods remains a topic of debate, as each has its own advantages and potential complications, and the current literature presents inconclusive results regarding their efficacy and safety profiles.

We found a systematic review that included studies up to 2023, where several important limitations were reported.^5^ This review failed to incorporate all available studies comparing K-wire fixation and screw fixation for pediatric lateral condyle fractures. Additionally, several new studies have been published since then, providing updated data that may potentially alter the previously observed conclusions.^5^ Therefore, the present systematic review and meta-analysis aims to provide a more comprehensive, updated, and methodologically rigorous synthesis of the comparative outcomes between K-wire fixation and cannulated screw fixation. Our primary objective is to assess the functional aspect and complication rates associated with each technique. We also explore the influence of patient age and fracture classification on treatment outcomes.

## MATERIALS AND METHODS

This systematic literature review was conducted according to the PRISMA guidelines *(Preferred Reporting Items for Systematic Reviews and Meta-Analyses)*.^6^ The main objective is to compare the clinical and functional outcomes of fixation with K-wires versus fixation with screws in the treatment of lateral condyle humeral fractures in children. The study population consisted of patients under 18 years of age diagnosed with lateral condyle humeral fractures. The intervention of interest was fixation using Kirschner wires, compared to fixation with screws. The primary outcomes assessed were functional recovery and postoperative infection rates. The secondary outcomes included lateral overgrowth, nonunion or malunion, and avascular necrosis (AVN) after surgical treatment. The included study designs were randomized clinical trials (RCTs) and cohort studies (prospective or retrospective).

This review was prospectively registered in the International Prospective Register of Systematic Reviews (PROSPERO) under registration number CRD420251012187. The review protocol, including eligibility criteria and outcome definitions, was established and submitted before initiating the systematic database search.

### Eligibility criteria

The eligibility criteria encompassed full-text RCTs published in English from inception to April 2025 and indexed in PubMed, Scopus, Embase, and Cochrane CENTRAL. The studies directly compared fixation with K-wires and screws in pediatric patients with lateral condyle humeral fractures and reported relevant clinical outcomes, including functional recovery and postoperative complications.

We excluded articles not available in full text via institutional or open-access platforms, conference abstracts, preprints, letters to the editor, case reports, case series, reviews, cross-sectional studies, and case-control studies. Studies that did not report sufficient information on the outcomes of interest were also excluded. In cases of duplicate publication, only the most complete and recent version of the study was retained for analysis.

### Search strategy

For the database search, descriptors related to "Lateral Condyle of the Humerus," "Fracture," "Screw Fixation," "K-wire," and "Children" were used. The descriptors were obtained from the *Medical Subject Headings* (MeSH), accessed at www.ncbi.nlm.nih.gov/mesh/. The Boolean operators "AND" and "OR" were used for term searches on the mentioned platforms, adhering to the articles’ inclusion and exclusion criteria.

### Study selection

Two independent reviewers, blind to each other's evaluations, jointly screened the titles and abstracts of all retrieved articles to identify those that met the predefined inclusion criteria. Studies deemed potentially eligible were then read in full to confirm their inclusion in the review. In cases of disagreement, a senior reviewer, who had access only to the disputed articles, made the final decision. The selection of studies was conducted using the Rayyan application.^7^ To ensure comprehensive coverage of the available literature, a snowballing strategy^8^ was also employed: the reference lists of all relevant systematic reviews identified through the initial search were screened to identify additional eligible primary studies, and the references of the included full-text articles were likewise reviewed after full reading to detect any potentially overlooked studies in the database search.

### Data synthesis

Data extraction was performed independently and in duplicate to ensure accuracy and reliability. Two reviewers extracted data from the included studies using a pre-designed data extraction form created in Microsoft Excel^®^ (2025 version). The form captured comprehensive details about the study characteristics, including population size, specifics of the intervention and control groups, methodological approaches, and the outcomes assessed. Any discrepancies between the reviewers were resolved through discussion or consultation with a senior reviewer to reach a consensus.

### Quality assessment

The risk of bias was assessed using two Cochrane tools, selected according to the study design: cohort studies were evaluated with the ROBINS-I *(Risk Of Bias In Non-randomized Studies of Interventions)* ^9^, while RCTs were assessed using the revised RoB 2 tool *(Risk of Bias tool for randomized trials).* ^10^ All assessments were conducted independently by two reviewers, and any disagreements were resolved through discussion and consensus. Publication bias was assessed using a funnel plot with enhanced contours, which displays point estimates against study weights and highlights areas of statistical significance to aid interpretation of the asymmetry.^11^

### Statistical analysis

Functional and complication outcomes, given their event-based nature, were analyzed using relative risks (RRs), with corresponding 95% confidence intervals (CIs). Heterogeneity among studies was assessed using Cochran's Q test and the I² statistic. A p-value < 0.10 and I² > 25% were considered indicative of significant heterogeneity. A random-effects model was applied to account for potential methodological variability among studies, despite statistical heterogeneity. The Restricted Maximum Likelihood (REML) method was the primary estimator for variance among studies. However, for the infection outcome, the REML method failed to converge due to the data characteristics; therefore, the Der Simonian and Laird model estimator was employed as an alternative.

Subgroup analyses were conducted based on study design (RCTs vs. cohort studies). A sensitivity analysis was conducted to assess the influence of individual studies on the pooled estimates. Although pre-specified in the protocol, the sensitivity analysis, which included only studies with low risk of bias, was not performed because none of the included studies met this criterion. Meta-regression analyses were conducted to explore the impact of two study-level covariates: mean patient age and the proportion of participants classified as Milch type II. These regressions aimed to investigate potential moderators of effect size. All statistical analyses were performed using RStudio (version 764), employing the ‘meta’ and ‘metafor’ packages.

## RESULTS

**Figure 1:**
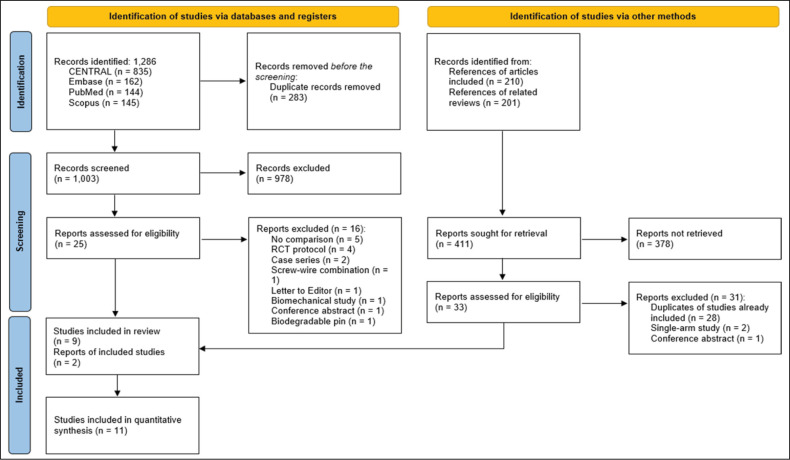
PRISMA flow diagram of the study selection and inclusion process.

A comprehensive search of the literature across multiple databases initially yielded 1,286 records. After the removal of duplicates, 1,003 unique articles remained for title and abstract screening. Based on the predefined eligibility criteria, 25 studies were selected for full-text review, resulting in the inclusion of 9 articles.^12–20^ To complement the database search, a search was conducted using the reference lists of the included studies and six additional systematic reviews on the same topic^5,21–25^, thereby identifying 411 additional records. After screening these, 33 full-text articles were reviewed, of which 2 additional studies met the inclusion criteria^26–27^. In total, 11 studies were included in the systematic review and meta-analysis.^12–20,26,27^ The complete study selection process, including reasons for exclusion, is detailed in Figure 1.

### Characteristics of the studies

Of the eleven included studies^12–20,26,27^, four were open-label randomized clinical trials^12,19,26,27^ and seven were retrospective cohort studies^13–18,20^. None of the trials were blinded. The study populations consisted of children with lateral condyle humeral fractures, typically indicated for surgery when displacement exceeded 2 mm, which was the common inclusion criterion across all studies. In total, 1,820 patients were included, of whom 514 were treated with cannulated screws and 1,306 with K-wires. Males represented approximately 51.15% of the total sample; however, two studies did not specify the sex distribution of their participants.^14,16^ The average age of the patients was approximately 7.8 years. The follow-up periods varied among the studies, ranging from 10 weeks to 4.8 years (on average), with seven studies reporting at least 12 months of follow-up.^12–15,20,26,27^ More details about the included studies are provided in Table 1.

**Table 1:** The main characteristics of the included studies.

Authors/Year	Country	Study Design	Follow-up	Included Patients		Mean Age (years)*		Gender†		Milch Classification	
				Screw	Kirshner Wire	Screw	Kirshner Wire	Screw	Kirshner Wire	Screw	Kirshner Wire
Afaque and Singh 2020	India	Open trial	18 mo‡	21	19	7.5a	7.9a	15 / 8	13 / 4	Type I: 8 Type II: 13	Type I: 7 Type II: 12
Cummings et al. 2023	United States	Retrospective cohort	12 mo	209	553	7.81 ± 3.61	8.78 ± 3.63	350 / 203	126 / 83	Type I: 102b Type II: 646	
Ganeshalingam et al. 2018	Canada	Retrospective cohort	4.8 y‡	101	235	7.15 ± 3.17	5.84 ± 2.77	62 / 39	143 / 92	Type I: 11 Type II: 89	Type I: 80 Type II: 154
Gilbert et al. 2016	United States	Retrospective cohort	6.8 mo‡	41	43	6.2 ± 3.03	5.2 ± 2.33	29 / 12	30 / 13	-	-
Gupta et al. 2023	India	Open trial	12 mo	23	23	6.26a	6.87a	15 / 8	19 / 4	Type I: 7 Type II: 16	Type I: 5 Type II: 18
Li et al. 2011	China	Retrospective cohort	39.4 mo‡	32	30	7.02a	6.83a	18 / 14	24 / 6	Type I: 9 Type II: 23	Type I: 5 Type II: 25
Pace et al. 2018	United States	Retrospective cohort	10 w‡	14	318	-	-	-	-	-	-
Stein et al. 2017	United States	Retrospective cohort	7.3 mo‡	26	22	5.9 ± 2.73	5.1 ± 1.73	20 / 6	13 / 9	Type I: 0 Type II: 26	Type I: 0 Type II: 22
Thapa et al. 2019	Nepal	Open trial	12 mo	23	23	6.26a	6.87a	15 / 8	19 / 4	Type I: 7 Type II: 16	Type I: 5 Type II: 18
Vergara and Fretes 2023	Paraguay	Open trial	6 mo	11	19	7.27 ± 2.80	6.42 ± 1.50	7 / 4	13 / 6	Type I: 5 Type II: 6	Type I: 7 Type II: 12
Wendling-Keim et al. 2020	Germany	Retrospective cohort	18 mo	13**	21	6.91 ± 2.83	5.67 ± 3.47	-	-	-	-

Legend: y = years; mo = months; w = weeks.

* Mean ± Standard Deviation;

** 4 patients in the group used screw + K wire;

† Male/Female;

‡ The authors reported the average follow-up; aThe author did not provide the standard deviation; bThe author did not stratify the classification for the groups.

### Metanalysis of function

A total of seven studies evaluated postoperative functional outcomes in the included patient population.^12,17–20,26,27^ However, only five were eligible for inclusion in the meta-analysis, as they used the same measurement tool, the Hardacre criteria.^12,17,18,20,26^ This scale categorizes functional recovery into four levels: excellent, good, fair, and poor, based on criteria such as pain, range of motion, and return to activity. For the purposes of this meta-analysis, only patients classified as having "excellent" function were considered to have experienced a positive outcome.

**Figure 2:**
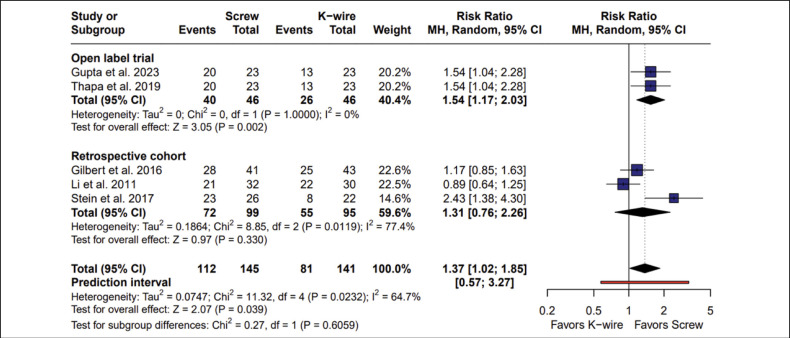
Forest plot showing the risk ratio for functional outcome based on the proportion of patients classified as "excellent" according to the Hardacre criteria, comparing cannulated screws with K-wires.

In total, 286 patients were included in the analysis. The pooled results demonstrated a favorable association for the cannulated screw group (RR = 1.37; 95% CI = 1.02 to 1.85; PI = 0.57 to 3.27; p = 0.606; I² = 64.7%) (Figure 2). Although the association suggests a benefit of cannulated screws, the wide prediction interval and moderate-to-high heterogeneity suggest that future studies could modify the overall conclusion. The sensitivity analysis revealed that this association remained statistically significant only when the study by Li et al. was excluded.

**Figure 3:**
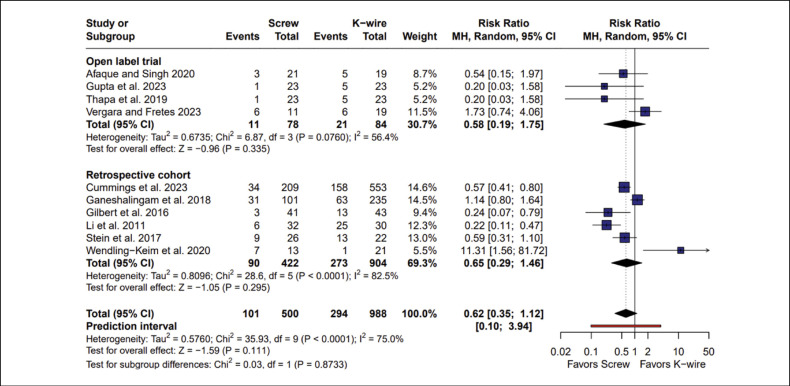
Forest plot showing the risk ratio for overall complication rates in patients treated with cannulated screws versus K-wires.

Meta-regression analyses did not show a significant relationship between functional outcome and the average age of participants (β = −0.32, p = 0.27) or the proportion of type II Milch fractures (β = 0.02, p = 0.25). Residual heterogeneity remained high, with a significant test for unexplained variance, suggesting that other unmeasured covariates may contribute to variability in functional outcomes. More details of the meta-regression are presented in Figure 3.

### Metanalysis of complications

The included studies reported postoperative complications inconsistently. Overall, the most commonly assessed and frequently observed complications were infection, malunion, nonunion, lateral overgrowth, and reduced range of motion. Less frequently reported complications included partial loss of fixation, nerve injury, and various adverse events. No serious complications or patient deaths were reported in any of the included studies.

**Figure 4:**
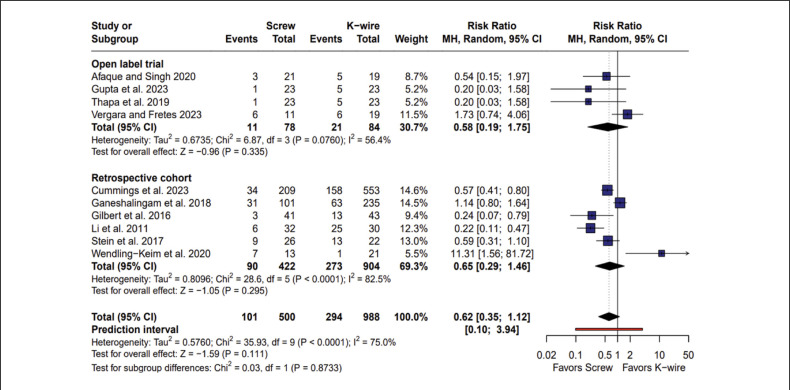
Forest plot showing the risk ratio for overall complication rates in patients treated with cannulated screws versus K-wires.

A general meta-analysis was conducted, grouping all reported complications, regardless of type. A total of 1,488 patients were included. The study by Pace et al. (2018) was excluded from this analysis, as the authors did not stratify the total number of complications by treatment group, reporting only the non-union rates separately. No statistically significant difference was observed between the groups regarding the overall risk of complications (RR = 0.62; 95% CI = 0.35 to 1.12; PI = 0.10 to 3.94; p = 0.111; I² = 75.0%) (Figure 4).

The meta-regression analyses did not show a significant association between complication rates and the average age of study participants (β = −0.12, p = 0.72) or the proportion of Milch type II fractures (β = −0.01, p = 0.60). Residual heterogeneity remained high, and the significant test for unexplained variance suggests that other unmeasured covariates may be influencing the observed results. More details of the meta-regression analysis are provided in Table 2.

**Table 2:** Meta-regression analysis of the relationship between covariates (average age and proportion of Milch type II) and outcomes (function and complications).

	Effect estimates	P-value	I (%)	Residual heterogeneity test
**Average Age (Function)**				
Intercept Average Age	2.31 −0.32	0.21 0.27	65.83	P=0.0300
**Proportion of Milch type II (Function)**				
Intercept Milch type II	-1.40 0.02	0.37 0.25	74.07	p = 0.0175
**Middle Ages (Complications)**				
Intercept Average Age	0.33 −0.12	0.88 0.72	79.73	p < 0.0001
**Milch type II ratio (Complications)**				
Intercept Milch type II	0.12 −0.01	0.92 0.60	78.02	p < 0.0016

### Infection

**Figure 5:**
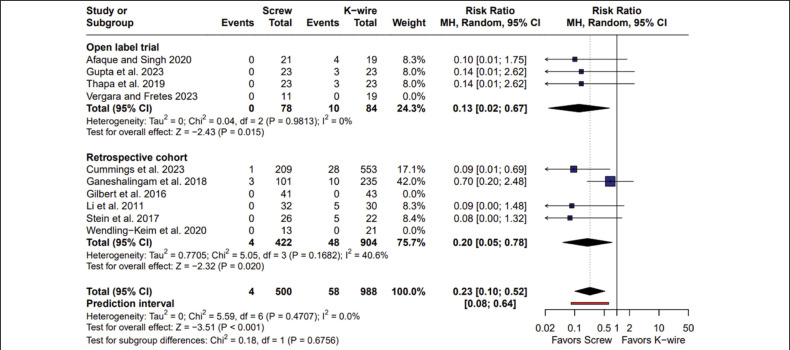
Forest plot showing the hazard ratio for infection in patients treated with cannulated screws versus K-wires.

A total of 1488 patients were included in this analysis, which primarily involved infections managed conservatively with antibiotic therapy only. Only a few patients required surgical reinterventions; however, stratified data distinguishing between conservative and surgical management were reported in few studies. The remaining studies provided only overall infection rates, preventing a stratified meta-analysis. The risk of infection was significantly lower in the group treated with cannulated screws (RR = 0.23; 95% CI = 0.10 to 0.52; PI = 0.08 to 0.64; p < 0.001; I² = 0%) (Figure 5). The narrow prediction interval suggests that future studies are unlikely to substantially alter these results.

### Lateral overgrowth

**Figure 6:**
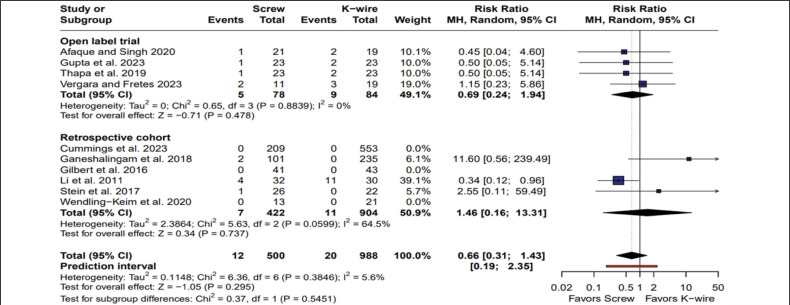
Forest plot showing the hazard ratio for lateral overgrowth in patients treated with cannulated screws versus K-wires.

Lateral overgrowth in 1488 patients was observed through clinical evaluation and radiographic comparison of the affected limbs. The outcome was typically identified by asymmetry in the length or alignment of the operated limb in follow-up images and physical examination, as reported by the included studies. No statistically significant difference was observed between the groups (RR = 0.66; 95% CI = 0.31 to 1.43; PI = 0.19 to 2.35; p = 0.295; I² = 5.6%) (Figure 6).

### Mobility reduction

**Figure 7:**
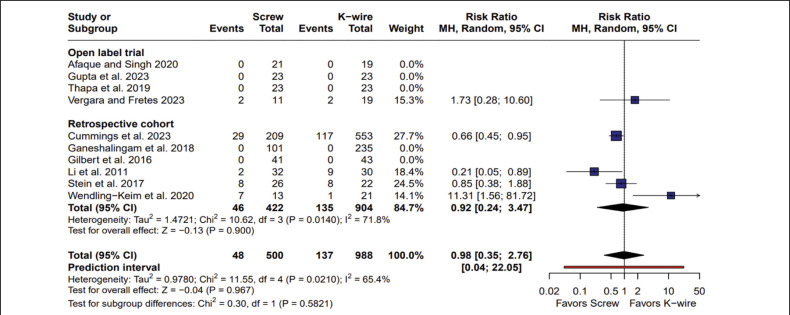
Forest plot showing the risk ratio for reduced range of motion in patients treated with cannulated screws versus K-wires.

The reduction in mobility was assessed in 1448 by measuring the postoperative range of motion (ROM) of the affected elbow, typically during follow-up after bone consolidation. These measurements were compared both to the expected physiological ROM for the pediatric population and to the ROM of the contralateral, unaffected limb. No statistically significant difference was found between the groups (RR = 0.98; 95% CI = 0.35 to 2.76; PI = 0.04 to 22.05; p = 0.967; I² = 65.4%) (Figure 7).

### Non-union and vicious consolidation

**Figure 8:**
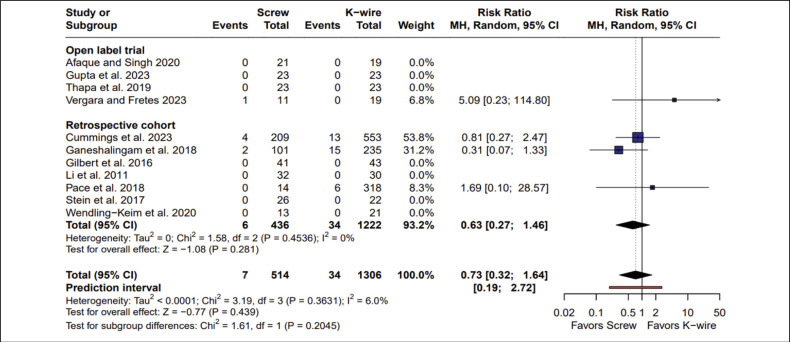
Forest plot showing the risk ratio for non-union/vicious consolidation in patients treated with cannulated screws versus K-wires.

A total of 1,820 patients were included in this analysis. Non-union and vicious consolidation were typically assessed through a combination of clinical and radiographic criteria during follow-up. This included evidence of incomplete or delayed bone consolidation on imaging, persistent pain or dysfunction, angular deformity, or the need for surgical revision due to inadequate fracture consolidation or alignment. In the combined analysis, no statistically significant difference was observed between the groups (RR = 0.73; 95% CI = 0.32 to 1.64; PI = 0.19 to 2.72; p = 0.439; I² = 6.0%) (Figure 8).

### Risk of bias of included studies

**Figure 9:**
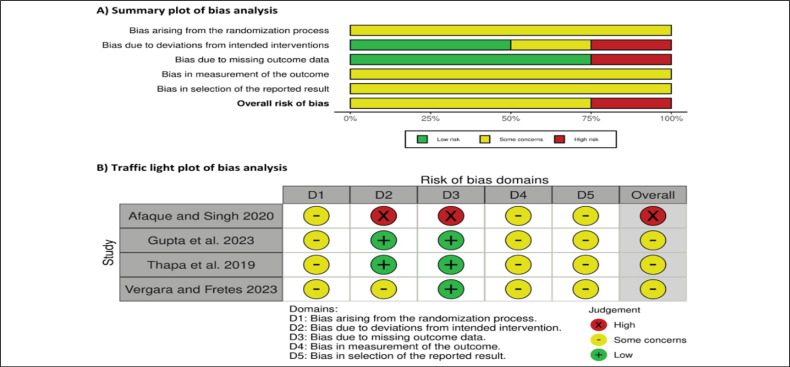
Assessment of the risk of bias of the included randomized clinical trials using the RoB 2.0 tool.

**Figure 10:**
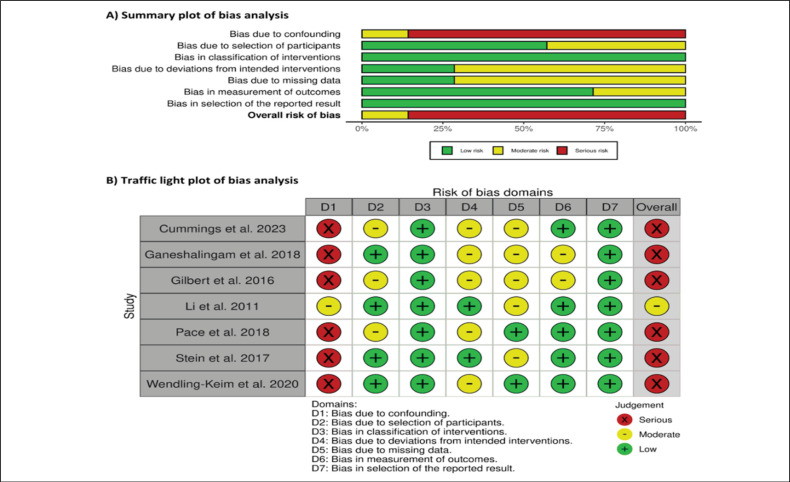
Assessment of the risk of bias in the included non-randomized studies using the ROBINS-I tool.

The risk of bias of the included studies is summarized in Figures 9 and 10.

A) Summary plot of the bias analysis. B) Traffic light plot of the bias analysis.

A) Summary plot of the bias analysis. B) Traffic light plot of the bias analysis.

### Risk of bias in randomized studies

All four studies raised some concerns regarding the randomization process (Domain 1). Although some authors described the use of random number tables and opaque envelopes to allocate patients, the details on whether the allocation sequence was concealed were not sufficiently reported, leaving the possibility of selection bias.^12,19,26^ In the study by Afaque and Singh (2020), the methodological information regarding this process was particularly limited. Regarding deviations from intended interventions (Domain 2), most studies adhered to the allocated treatments^12,19,26^, but in the case of Afaque and Singh (2020), patients without adequate follow-up were excluded from the assessment, and no intention-to-treat analysis was applied, which increased the risk of bias in this domain. Concerning missing outcome data (Domain 3), most studies had complete follow-up or minimal loss, while Afaque and Singh (2020) excluded approximately 15% of participants without accounting for these losses in the analysis. In Domain 4, related to outcome measurement, none of the studies blinded their assessors, and all used subjective measures such as pain and functional scores, leading to some general concerns.^12,19,26,27^ In Domain 5, none of the included trials had a published protocol or registered analysis plan, which hindered verification of outcome reporting decisions and contributed to concerns in all cases.^12,19,26,27^

### Risk of bias in cohort studies

The most frequent and serious concerns were related to confounding factors (Domain 1). Six studies were classified with serious risk in this domain due to allocation driven by institutional practice, time, or clinical severity at presentation. The study by Li et al. (2011) was considered to have a moderate risk, mainly due to treatment choice likely influenced by the surgeon's preference or fragment morphology. In Domain 2 (participant selection), most studies were classified as low risk, although moderate concerns were identified in three cases where inclusion was limited to patients with complete follow-up, potentially excluding more severe or complex cases.^13,15,16,20^ For missing outcome data (Domain 3), all studies were considered low risk, with no substantial attrition or differential follow-up.^12–20,26,27^ In deviations from intended interventions (Domain 4), moderate risk was presented in four studies due to limitations in describing whether all patients received the initially planned technique or if intraoperative decisions altered the intervention.^13–16,18^ In bias due to handling of missing data (Domain 5), moderate concerns were observed in five studies that lacked detailed information on the impact of incomplete follow-up or did not conduct appropriate sensitivity analyses.^13,15–18^ For Domain 6 (outcome measurement), most studies were low risk, although Ganeshalingam et al. (2018) and Gilbert et al. (2016) raised moderate concerns due to the lack of blinding and inconsistent assessment methods. All studies were judged to be at low risk in Domain 7 (selection of reported outcomes).^12–20,26,27^

## DISCUSSION

This systematic review with meta-analysis demonstrated a statistically significant association between cannulated screw fixation and superior functional outcomes compared to K-wires in pediatric lateral condyle humeral fractures. Additionally, screw fixation was associated with a significantly lower risk of postoperative infection, while no significant difference was identified between the two techniques in overall complication rates, lateral overgrowth, range of motion reduction, or nonunion and malunion. These findings highlight potential advantages of screw fixation, particularly in reducing superficial infections, but also emphasize the need for cautious interpretation of functional outcomes.

Stable fixation with cannulated screws allows for early mobilization, potentially reducing the risk of joint stiffness and facilitating a quicker return to function. In contrast, fixation with K-wires often requires prolonged immobilization due to less rigid stabilization, which may contribute to higher rates of elbow stiffness and superficial infections, as the wires remain exposed through the skin and can serve as entry points for pathogens.^13^ This heterogeneity suggests that, although the average effect favors screw fixation, the results of individual studies vary, and in some cases, fixation with K-wires may produce comparable outcomes. The broad PI highlights the uncertainty and potential influence of factors such as surgical technique, patient age, fracture pattern, and postoperative rehabilitation protocols on the effectiveness of the fixation method. Therefore, although current evidence leans towards the benefits of screw fixation, clinicians should interpret these findings with caution, considering the specific clinical context and patient characteristics.

We conducted meta-regression analyses to explore potential sources of heterogeneity, with particular focus on patient age and fracture classification. These factors are important in the prognosis of pediatric fractures. Younger children may have different healing capacities and risks than older children, and the degree of fracture displacement may affect treatment decisions and outcomes. However, our analyses did not identify a significant moderating effect of age or fracture severity on functional outcomes. This suggests that the observed heterogeneity in functional outcomes may be attributed to other unmeasured variables, such as variations in surgical technique, postoperative care protocols, or patient adherence to rehabilitation. Therefore, although our meta-regression did not point to specific factors influencing variability, it highlights the complexity of treatment outcomes and the need for individualized clinical assessments.

Previous systematic reviews have also explored comparative functional outcomes between Kirschner wire fixation and screw fixation in pediatric lateral condyle fractures. The study by Haghighi et al. (2025) reported a slightly higher rate of excellent functional outcomes in the screw group (82.2%) compared to the K-wire group (75.3%) using Hardacre criteria, suggesting a favorable trend towards screw fixation. Similarly, Cho et al. (2023)^5^, in a meta-analysis including a randomized clinical trial and three cohort studies, found a significantly lower risk of postoperative range of motion limitation in the screw group (RR = 3.75; 95% CI: 1.54–9.18; P < 0.01), reinforcing the notion of superior functional recovery with screws. The work of Birkett et al. (2020) also demonstrated higher rates of excellent outcomes with screw fixation (95%) than with K-wire fixation (86%), although their analysis was narrative due to heterogeneity in outcome measures. In contrast, our systematic review included a larger set of studies and patients and applied a more comprehensive, statistically rigorous approach. By incorporating the prediction interval (PI), we were able to quantify the expected variability in functional outcomes in future scenarios, revealing that the effect size favoring screws, while significant, is not universally consistent. Furthermore, through meta-regression analyses, we investigated potential moderators such as patient age and Milch classification, enhancing the depth of our interpretation. These advanced methods enabled a more cautious, context-sensitive understanding of functional outcomes, going beyond simple aggregate effects and addressing heterogeneity among studies that previous meta-analyses did not adequately explore.

Several previous systematic reviews assessed the complication profiles associated with K-wire and screw fixation. The article by Sinha et al. (2023) found no statistically significant differences in overall complication rates between the two methods, reporting complications in 23.7% of K-wire cases and 20.4% of screw fixations, although union was more common in the screw group (93.2% vs. 91.1%). Similarly, Eckhoff et al. (2022) observed high union rates across all techniques (> 99%) in their comparison of K-wire under the skin and K-wire through the skin and screws. They highlighted that the K-wires, especially when not kept under the skin, had a shorter union time and better postoperative range of motion, although with higher rates of superficial infection. Tan et al. In 2018, in a review of 2,440 pediatric cases, they identified a wide spectrum of complications, such as valgus/varus deformities, loss of motion, prominence of the lateral condyle, and osteonecrosis. Infection occurred in approximately 4.8% of cases, and pain caused by the implants occurred in 22% of cases, regardless of fixation type. When compared with our systematic review, these findings were consistent in showing no major differences in overall complication rates. We found a significantly lower risk of postoperative infection in the screw group, while other complications, such as limited range of motion, non-union, and lateral overgrowth, did not differ significantly.

Regarding postoperative infection, most studies reported higher rates with the use of K-wires, especially when the wires were left outside the skin.^5,21,22^ Our work corroborated this association by demonstrating a significantly lower risk of infection with screw fixation. Exposed K-wires through the skin create a direct pathway for bacterial entry, increasing the risk of contamination.^21^ These factors, together, help explain why screw fixation consistently shows better outcomes in terms of infection prevention.

### Limitations of the study

This study has some limitations that must be acknowledged. Most of the included studies were retrospective cohort designs, which are inherently susceptible to selection bias and confounding factors, particularly regarding treatment allocation based on institutional protocols or surgeon preference. Secondly, the definitions of outcomes, especially for complications such as lateral overgrowth, vicious consolidation, and reduced range of motion, were not fully standardized across studies, which may introduce measurement bias and limit the comparability of results. Although subgroup analyses and meta-regression were performed to explore heterogeneity, several potentially relevant covariates, such as the exact surgical technique, duration of postoperative immobilization, or time to hardware removal, were not consistently reported and could not be analyzed. Furthermore, the inability to blind outcome assessors in all included trials and the absence of intention-to-treat analyses in at least one study may have inflated effect estimates. Finally, although publication bias was formally assessed, the limited number of studies for some outcomes may reduce the power of funnel plot analysis, and the observed asymmetry in the complication outcome suggests that reporting bias cannot be entirely ruled out.

## CONCLUSION

Based on the current body of evidence, cannulated screw fixation appears to be associated with better functional outcomes and a lower risk of infection than Kirschner wire fixation for the management of lateral condyle fractures in children. However, the observed functional benefit may change as new evidence emerges. No significant difference was identified between the techniques regarding other postoperative complications. These findings should be interpreted with caution due to the predominantly observational nature of the included studies. Therefore, while screw fixation may offer advantages in clinical contexts where infection prevention is a priority, the current evidence does not support its overall superiority.

## Data Availability

The underlying content of the research text is contained in the manuscript.
